# Cellular imaging: a key phenotypic screening strategy for predictive toxicology

**DOI:** 10.3389/fphar.2015.00191

**Published:** 2015-09-08

**Authors:** Jinghai J. Xu

**Affiliations:** Merck and Co.Kenilworth, NJ, USA

**Keywords:** hepatotoxicity, cardiotoxicity, genetic toxicology, cellular imaging, predictive toxicology, discovery toxicology, phenotypic screening

## Abstract

Incorporating phenotypic screening as a key strategy enhances predictivity and translatability of drug discovery efforts. Cellular imaging serves as a “phenotypic anchor” to identify important toxicologic pathology that encompasses an array of underlying mechanisms, thus provides an effective means to reduce drug development failures due to insufficient safety. This mini-review highlights the latest advances in hepatotoxicity, cardiotoxicity, and genetic toxicity tests that utilized cellular imaging as a screening strategy, and recommends path forward for further improvement.

## Introduction

Modern medicines have saved countless lives. One needs only to trace the discovery of antiretroviral drugs to marvel at its contribution to human health (Broder, 2010). However, new therapies are necessary to address still unmet medical needs while adhering to the timeless adage of “first doing no harm” to patients. Predictive toxicology meets the latter challenge by offering accurate and timely predictions to minimize drug toxicity (Xu and Urban, 2010). It is recognized as a frontier for identifying better medicines in a more cost-effective manner by biopharmaceutical research and regulatory communities (FDA, 2010).

To successfully practice predictive toxicology, one must adopt an integrated approach utilizing innovations from multiple fields of science and engineering. In recent years there is a resurgence of phenotypic screens in drug efficacy discovery (Moffat et al., 2014). Likewise to improve confidence in the translatability of toxicity predictions, one should also incorporate phenotypic screens as a key strategy. This is because the “phenotype” of a specific safety signal can be caused by many underlying mechanisms and pathways, thus a reductionist “one gene/protein/mechanism to one toxicity” approach most likely will not be sufficient. This does not mean that one should revert to simple cell death measurements. Instead, more specific cellular functions that are vital to sustain physiological homeostasis can serve as translational links between non-clinical tests and clinical observations. Among many practical choices of phenotypic screens, cellular imaging technologies can be applied as “phenotypic anchors” to identify the same histopathology that occurs *in vivo*. Cellular imaging encompasses the techniques that allow quantitative detection and measurement of cellular structures and components including organelles and biomolecules ranging from macromolecules to small ions, often aided by automated microscopes, digitized cameras, and image analysis algorithms. Since “seeing is believing,” this “phenotype first” approach assures an unbiased study for compounds that affect important cellular homeostasis without exhausting all possible mechanistic tests. Of course, “phenotype first” does not preclude mechanistic investigations to better understand the underlying reason(s) of drug safety findings. Often it is a holistic approach relying on mechanism-informed phenotypic test, coupled with pharmacokinetic and pharmacodynamics (PKPD) modeling that produces the best overall prediction toward clinical outcome.

## Hepatic toxicity imaging

Hepatic toxicity ranked highest in research priorities among adverse drug reactions in the pharmaceutical industry, and current detection systems remain imperfect especially with regard to idiosyncratic hepatotoxicity (Opar, 2012). Key mechanisms of drug-induced liver injury (DILI) include oxidative stress and/or mitochondrial damage leading to apoptosis or steatosis, and hepatobiliary transporter inhibition leading to cholestasis (Kaplowitz, 2005; Tujios and Fontana, 2011).

Mechanism-informed phenotypic tests using cellular imaging have been developed to encompass these key DILI mechanisms. For example, a panel of fluorescent imaging probes has been applied to measure oxidative stress, mitochondrial function, glutathione content, and hepatocellular lipidosis simultaneously in primary human hepatocytes (Xu et al., 2008). It has demonstrated ~60% sensitivity and ~95% specificity for drugs that have caused idiosyncratic DILI. This hepatocyte imaging assay technology has been successfully applied to both differentiate compounds for the same pharmacologic target (e.g., p38 MAP kinase inhibitors), and study the mechanism of DILI signals observed in the clinic (e.g., Her2 receptor antagonist) (Xu et al., 2011). A similar imaging approach measuring oxidative stress in primary human hepatocytes resulted in 41% sensitivity and 86% specificity, and was shown to have better predictivity than HepG2 cell lines or HepG2 plus hepatic metabolism from the liver S9 fractions (Garside et al., 2013). These studies demonstrated the application of cellular imaging technology to predict DILI, and highlighted the need to enhance test sensitivity by recapitulating additional DILI mechanisms and pathways.

Since inhibition of a panel of hepatobiliary transporters is another known pathway of drug-induced cholestasis (Morgan et al., 2013), cellular imaging was applied to assess transporter functions. The assay utilized quantitative imaging of fluorescent bile acid and its disposition in the bile canaliculi compartment of the hepatocyte cultures (Xu et al., 2011). As protein trafficking and sorting disturbances also play an integral part of intrahepatic cholestasis (Hayashi and Sugiyama, 2013), whole cell systems as opposed to isolated membrane vesicles over-expressing one transporter at a time should continue to play a key role in phenotypic screening of cholestasis-inducing drugs.

Several improvements on these cellular imaging tests should be explored to further enhance accurate prediction of DILI:

1. Long-term: Adopt a longer-term test system that maintains differentiated metabolic functions of human liver. Recently with advancement in iPSC-derived hepatocyte cultures, multi-day testing appeared promising. However, these longer-term models still need to be assessed with more DILI negative drugs to fully assess specificity (Ware et al., 2015).
2. Diversity: Apply more than one human liver origin with defined genetic background to increase test sensitivity toward idiosyncratic DILI. Patient-derived hepatic stem cells that can be differentiated into adult hepatocyte cultures can be interesting models to explore idiosyncratic causes of DILI.
3. Probes: Expand fluorescent imaging probes to include additional bile acid analogs that are substrates of both NTCP and BSEP transporters, in addition to cholyl lysyl fluoresceine which is a more specific substrate for OATP/MRP transporters (de Waart et al., 2010).
4. Inflammatory and multi-cell systems: Explore the role of pro-inflammatory cytokines and/or Kupffer cells for possible synergistic effects in test sensitivity without sacrificing specificity (Cosgrove et al., 2009), and assess the potential benefit of 3D liver chip with controlled microfluidics.
5. Modeling: Mechanism-based PKPD modeling approaches should be applied to integrate any *in vitro* measurements into a holistic *in vivo* prediction (Woodhead et al., 2014). Predictions made by the more complex model should also be compared to simple *in vitro*-*in vivo* scaling or classification approaches based on exposure multiples or pre-defined safety margins.

## Cardiac toxicity imaging

Adverse cardiovascular effects ranked highest among safety reasons for delayed approvals or non-approvals by the US Food and Drug Administration (FDA) between 2000 and 2012 (Sacks et al., 2014). Many drugs that prolong the QT interval in electrocardiograms block the delayed rectifier K^+^ current (Ikr) by blocking one key ion channel, the hERG channel. However, not all hERG channel blockers cause QT interval prolongation nor translate to the more severe clinical concern, torsade de pointes [TdP, or twisting of the points in electrocardiograms (Vargas, 2010)]. So QT prolongation is a surrogate biomarker that is not very specific to TdP. In addition to TdP there are other mechanisms of cardiotoxicity. Cardiovascular functional abnormalities can also arise through impaired left ventricular function characterized by changes in cardiomyocyte contractility (Doherty et al., 2013). Therefore, a more holistic screening approach is needed. Since 2013, the FDA working with other international research communities have an on-going initiative to develop better preclinical tools to evaluate cardiovascular safety with the ultimate goal of preventing early stage compounds from being unnecessarily discontinued, while still screening out molecules that are either pro-arrhythmic or lead to changes in cardiomyocyte contractility (Sager et al., 2014).

Synchronously beating human cardiomyocytes should be explored as a key phenotypic screening model. Despite some limitations of iPSC-derived cardiomyocytes, they are the future for large-scale functional cardiotoxicity screening models in preclinical drug evaluation (Puppala et al., 2013). This is because there is a virtually unlimited supply of functionally competent cells, and there is the possibility to create cells from defined genetic background thus lending the model amenable to further mechanistic studies. Researchers have studied 131 drugs using iPSC-derived cardiomyocytes, using fast kinetic imaging-based Ca^2+^ flux as continuously cell-beating measurements (Sirenko et al., 2013). Each output (beat rate, peak characteristics, and cell viability) generated as a result of such cellular imaging assay has yielded a panel of imaging “signatures” for understanding the type of pathophysiological effect that a chemical may have on the heart. Not surprisingly, beat rate and several peak shape parameters were found to be better predictors than simple cell viability. In another study, video-based microscopic imaging recapitulated expected drug effects in stem-cell-derived cardiomyocytes without the use of exogenous labels and produced the same beat traces as patch clamp (Maddah et al., 2015). Recently, the throughput and accuracy of iPSC-based model to detect changes in cardiomyocyte contraction was found applicable in drug discovery screening, with a sensitivity and specificity of 87 and 70%, respectively (Pointon et al., 2015).

Tyrosine kinase inhibitors (TKIs) are efficacious against tumors harboring mutations and/or over-expressing such kinases. However, TKIs have been hampered by cardiotoxicity issues detected most frequently as changes in left-ventricular ejection fraction (LVEF), which were not predicted by hERG inhibition *per se*. Using multi-parameter cellular imaging it was demonstrated while crizotinib, sunitinib, and nilotinib disrupted the normal beat pattern of human iPSC-derived cardiomyocytes, erlotinib did not. These alterations in iPSC-cardiomyocyte beat pattern correlated well with known clinical cardiac outcomes of these drugs, demonstrating the potential to use this phenotypic screen as a routine test to identify safer compounds in a class of drug candidates (Doherty et al., 2013).

Several improvements on these cellular imaging screens should be explored to further enhance the overall prediction of cardiotoxicity:

1. beating disturbances by a variety of drugs with different cardiotoxic mechanisms
2. longer-term cellular models to study cardiac hypertrophy
3. drug metabolites by introducing hepatic metabolism (e.g., either hepatic S9 fraction or multi-cell systems)
4. more test qualification with large compound sets consisting of an equal number of positive and negatives that have been rigorously adjudicated. A balanced test set of compounds with more negative or clean compounds is critical to assess test specificity.

## Genetic toxicity imaging

Carcinogenicity remained a major nonclinical safety finding among FDA non-approvals and delayed approvals between 2000 and 2012 (Sacks et al., 2014). In one of the largest retrospective analyses of pharmaceutical compounds to date, it was demonstrated that significant R&D time, resources, and animal usage can be reduced by relying on the near 100% negative predictive values (NPV) of combined tests evaluating genetic toxicity, hormonal perturbation, and evidence of neoplasia in chronic rat toxicology studies (Sistare et al., 2011). To minimize the impact of carcinogenicity findings in late-stage drug development, both mutational and chromosomal changes need to be evaluated as they can be caused by different underlying mechanisms (FDA, 2012).

Cellular imaging has greatly enhanced the efficiency and accuracy of measuring both mutational and chromosomal changes. The Ames test remains a cost-effective stethoscope to study genetic toxicology (Claxton et al., 2010). In order to enable rapid scoring of mutant bacterial colonies, the Salmonella strains used in the Ames test were engineered to express bioluminescent proteins (Aubrecht et al., 2007). The high-throughput imaging based on automated counting of the number of surviving bioluminescent colonies offered a far more accurate assessment of genotoxicity than bulk biomass measurements (Xu and Aubrecht, 2010).

With regard to chromosomal changes, the *in vitro* micronucleus test is an accepted test by regulatory authorities (EMA and FDA) (FDA, 2012). While traditional micronucleus test relied on manual counting of the number of micronuclei in thousands of cells per treatment sample and hundreds of samples per test compound, cellular imaging has made this task more efficient. The increased throughput and accuracy of computerized analysis of micronuclei frequency enabled rapid structure-activity profiling of drug candidates during lead optimization (Xu and Aubrecht, 2010).

## Conclusion

From a systems perspective, since *in vitro* genotoxicity tests have near 100% negative predictive value toward *in vivo* genotoxicity outcome, are among the most cost-effective to operate (i.e., using bacterial strains and mammalian cell lines), and include both general cytotoxicity and cell growth inhibition as integral part of assay readouts, *in vitro* Ames and micronucleus should be employed as the foundational tests to select those “clean” compounds for further R&D investments. In the lead optimization stage of small-molecule drug discovery, these genotoxicity tests should be followed by cardiac and hepatic toxicity screening using primary cells or iPSCs next, then other organ-specific tests on an *ad hoc* basis last. The *ad hoc* tests are reserved for program-specific purposes, e.g., triggered by hypotheses about the mechanism-based toxicity or prior *in vivo* findings for previous compounds in the program. Examples of such *ad hoc* assays include: muscle, kidney, neural, teratonenic, developmental, myelotoxicity, and lymphotoxicity. The potential for multi-organ chip to improve toxicity prediction should also fall into this last category until its predictivity and throughput make it possible for front-loading (Bhatia and Ingber, 2014). The *ad hoc* assays can also include further investigation of previous positive findings, e.g., micronucleus positives can be imaged for other markers to differentiate chromosome breaks (clastogens) vs. chromosome loss (aneuploidy), where an acceptable safety margin can allow for further drug development (Cheung et al., 2014). In the complex business of defining drug safety, “clean” compound in all test systems are not always realistic. One should therefore use a balanced and holistic approach starting with defining an acceptable safety profile for a drug candidate, understanding the predictive value and limitation of each test system, and a “weight-of-evidence” approach integrating findings from human cells, *in vivo* animal testing, and human clinical trials. Table 1 summarizes the imaging tests and their predictive values described in this mini-review.

**Table 1:** **A summary of imaging tests and their predictive values reviewed by this article**.

**Cell model (reference)**	**Imaging technique**	**Threshold for positives**	**Predictive value**	**Example image**
Human hepatocyte (Xu et al., 2008)	Fluorescence imaging of oxidative stress, mitochondrial function, glutathione content and hepatocellular lipidosis	>2.5 fold above or < 2.5 fold below the vehicle control mean values (depending on which fluorescence channel). Appropriate cut-off levels were selected using ROC curves	~60% sensitivity and ~95% specificity	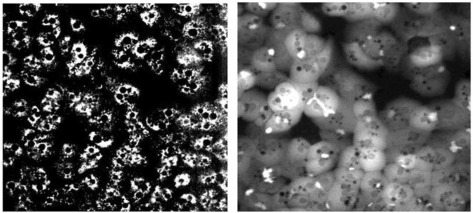
Human hepatocyte (Garside et al., 2013)	Fluorescence imaging of oxidative stress	>6 SD of the vehicle control mean values	41% sensitivity and 86% specificity	
Human hepatocyte (Xu et al., 2011)	Fluorescence imaging of bile acid and its disposition in bile canaliculi	< 2.5 fold below the vehicle control mean values	TBD	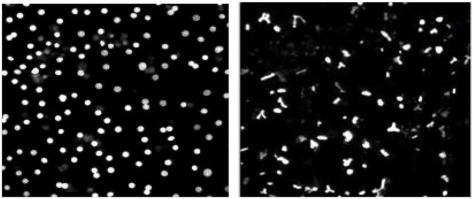
iPSC-derived human cardiomyocytes (Sirenko et al., 2013)	Fast kinetic imaging-based Ca^2+^ flux as continuously cell-beating measurements (beat rate, amplitude, and other beat parameters)	>1 SD of the vehicle control mean values	TBD	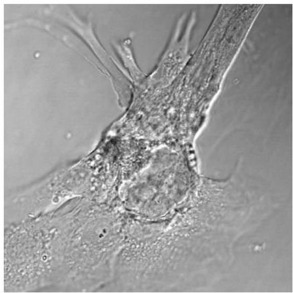
iPSC-derived human cardiomyocytes (Pointon et al., 2015)	Fast kinetic imaging-based Ca^2+^ flux as continuously cell-beating measurements (peak count, average peak amplitude, average peak width, average peak rise time, average peak decay time, and average peak spacing)	The median of the positive and negative control wells were set at 0 and −100, respectively, and the signals from all wells scaled to this range. Appropriate cut-off levels were selected using ROC curves	87% sensitivity and 70% specificity, using peak count	
Ames (Xu and Aubrecht, 2010)	Automated counting of the number of surviving bioluminescent colonies	Same as manual counting	100% combined negative predictive value to carcinogenicity test	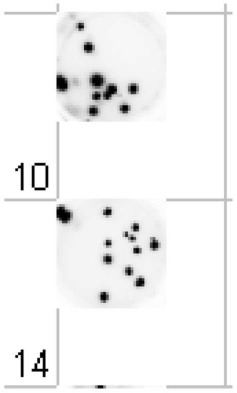
*In vitro* micronucleus (Xu and Aubrecht, 2010)	Automated scoring of the micronucleus frequency	Same as manual scoring	100% combined negative predictive value to carcinogenicity test	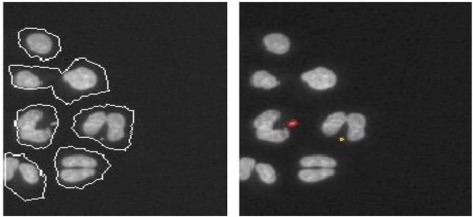

*SD, standard deviation; ROC, receiver operator characteristic; TBD, to be determined*.

Safety deficiencies account for more than half of delayed FDA approval or non-approval of new drug applications from 2000 to 2012 (Sacks et al., 2014). Predictive toxicology holds the key to reduce this attrition and make R&D investment more sustainable. To realize its promise practitioners of predictive toxicology must continue to integrate diverse innovations from multiple fields of biomedical engineering, including:

1. Predictive and reliable cellular models: esp., evaluation, characterization, production, and standardization of predictive human cells
2. Mechanism-informed phenotypic screening strategy, including cellular imaging as a key component as it provides a phenotypic anchor and direct linkage to *in vivo* histopathology
3. Quantitative therapeutic window predictions: e.g., using mechanism-based scaling or predictive PKPD modeling

One may envision a future when the banking of pluripotent stem cells from individual human donor with defined genetic makeup and subsequent expansion into fully-functional organ parenchymal cells as a desirable model toward predicting personalized drug responses, for both drug efficacy and safety. But before we achieve that dream, the holistic applications of an integrated approach as highlighted above have already marched us a long way toward transforming toxicology to improve product safety.

### Conflict of interest statement

The author is an employee of Merck and Co. However, views expressed in this mini-review are the author's own and do not reflect positions of any company or organization.
